# CRISPR-Cas adaptation in *Escherichia coli*

**DOI:** 10.1042/BSR20221198

**Published:** 2023-03-09

**Authors:** Damjan Mitić, Edward L. Bolt, Ivana Ivančić-Baće

**Affiliations:** 1 Department of Biology, Faculty of Science, University of Zagreb, 10000 Zagreb, Croatia; 2 School of Life Sciences, University of Nottingham, Nottingham NG7 2UH, U.K.

**Keywords:** adaptation, Cas1-Cas2, CRISPR-Cas, E. coli, RecBCD

## Abstract

Prokaryotes use the adaptive immunity mediated via the Clustered Regularly Interspaced Short Palindromic Repeats and CRISPR associated (CRISPR-Cas) system for protection against invading elements such as phages and plasmids. The immunity is achieved by capturing small DNA fragments or spacers from foreign nucleic acids (protospacers) and integrating them into the host CRISPR locus. This step of CRISPR-Cas immunity called ‘naïve CRISPR adaptation’ requires the conserved Cas1–Cas2 complex and is often supported by variable host proteins that assist in spacer processing and integration. Bacteria that have acquired new spacers become immune to the same invading elements when reinfected. CRISPR-Cas immunity can also be updated by integrating new spacers from the same invading elements, a process called ‘primed adaptation’. Only properly selected and integrated spacers are functional in the next steps of CRISPR immunity when their processed transcripts are used for RNA-guided target recognition and interference (target degradation). Capturing, trimming, and integrating new spacers in the correct orientation are universal steps of adaptation to all CRISPR-Cas systems, but some details are CRISPR-Cas type-specific and species-specific. In this review, we provide an overview of the mechanisms of CRISPR-Cas class 1 type I-E adaptation in *Escherichia coli* as a general model for adaptation processes (DNA capture and integration) that have been studied in detail. We focus on the role of host non-Cas proteins involved in adaptation, particularly on the role of homologous recombination.

## Introduction

Unusual repetitive sequences were first described in *Escherichia coli* in 1987 during analysis of the nucleotide sequence of the *iap* gene responsible for isozyme conversion of alkaline phosphatase [1]. Similar intriguing repeats were then observed in Archaea [2] and many other species. Repeats are short repetitive partially palindromic noncoding DNA sequences separated by short variable sequences known as spacers. These sequences were termed as clustered regularly interspersed short palindromic repeats (CRISPR arrays), and the first CRISPR-associated genes (*cas*), located in the vicinity of a CRISPR locus, were described in 2002 [3]. Widespread presence and evolutionarily conserved repeats suggested an important role for these sequences, but their exact biological role was enigmatic at the time [4]. Today, CRISPR-Cas systems have been found in ∼90% of archaea and ∼40% of bacteria [5]. The possibility that CRISPR-Cas functions as an adaptive immunity system was proposed when homologies between some spacers within a few CRISPR arrays and virus and plasmid sequences were discovered [6–8]. The initial idea was that RNA transcripts of spacers act on target recognition by a mechanism analogous to the RNA interference system used by eukaryotic cells [9]. The experiments done in the lactic bacteria *Streptococcus thermophilus* showed that bacteria that became resistant after the phage challenge have integrated new spacers derived from phage genomic sequences. Removal or addition of spacers into the CRISPR array restored sensitivity or established novel resistance, respectively. This led to the conclusion that the presence of a spacer in the CRISPR array identical to a phage sequence provides resistance against that phage [10].

Despite their common role in adaptive immunity, CRISPR-Cas systems are phylogenetically, organizationally, and functionally very diverse. According to the most recent classification which is based on a combination of phylogenetic, comparative genomic and structural analysis, CRISPR-Cas systems are divided into two classes (class 1 and class 2), six types (types I–VI), over 30 subtypes, and several highly derived CRISPR variants with still unknown functions [11]. The major difference between the classes is in the organization of the effector modules. Class 1 CRISPR-Cas systems include types I, III, and IV, which use a multiprotein effector complex comprises several Cas proteins, whereas class 2 CRISPR-Cas systems include types II, V, and VI, which use a single multidomain protein in which all enzymatic activities are combined. The adaptation module is relatively uniform across CRISPR-Cas systems and consists of Cas1 and Cas2 that form a structurally stable protein complex [5,12]. In many other systems (several sub-types within type I, II, and V) adaptation modules also include Cas4. Cas4 is a RecB-like nuclease that forms a complex with Cas1–Cas2 and is involved in selecting and acquiring protospacer-adjacent motif (PAM)-containing spacers [13–15].

The action of the CRISPR-Cas immunity is usually divided into three stages. Each of these stages is mediated by a distinct set of Cas proteins which are organized into functional modules [11]. Briefly, the process of integration of new spacers into the CRISPR array, called adaptation, is the first stage of the CRISPR-Cas immunity [16,17]. The second stage, expression and maturation of crRNAs, involves transcription and translation of *cas* genes and transcription of the CRISPR array into a long pre-CRISPR RNA that is processed into a functional mature CRISPR RNA (crRNA) [18,19]. One or more Cas effector proteins and crRNA assemble together into a ribonucleoprotein complex [18]. The crRNAs are then used as guides to recognise and cleave the target DNA or RNA during the interference stage by the effector proteins [18,20,21].

The molecular insights of spacer acquisition and elucidation of CRISPR-Cas immunity are studied in the greatest detail in *E. coli*, so we will focus the remainder of the review on the CRISPR-Cas class 1 type I-E adaptation. Adaptation mechanisms in *E. coli* represent an excellent basic model how CRISPR-Cas immunity is generated, updated and combined with other cellular processes. In *E. coli*, the adaptation is mediated by the Cas1–Cas2 complex [16,17], the processing of pre-crRNA is mediated by the CasE (or Cas6e) endonuclease which remains bound to the 3′ end of the mature crRNA [22,23]. The 61 nt long crRNA is then assembled with Cas proteins into the multimeric effector complex called Cascade (CRISPR-associated complex for antiviral defence) [18,24–26]. Using crRNA as a guide, the Cascade complex scans for target DNA and initiates complementary base pairing with the target DNA strand near the 5′ end of the crRNA (seed region), and base pairing then extends along the crRNA forming a structure known as the R-loop [24,26–28]. Complete formation of the R-loop induces a conformational change in the Cascade complex that recruits Cas3 [29–34]. The Cas3 nuclease-helicase is then allowed to bind to the R-loop and degrade DNA in a unidirectional ATP-dependent manner (reviewed in [35]). This stepwise activation mechanism of DNA degradation is quite unique among CRISPR-Cas systems and was suggested as a mechanism to minimize off-targeting [36]. Degradation of DNA interferes with virus or plasmid replication and confers immunity to the bacterial cell. More detailed information on the CRISPR-Cas adaptation in other CRISPR-Cas types and species that we didn't address here can be found in many excellent reviews [37–44].

## The role of PAM in CRISPR-Cas adaptive immunity

Bioinformatic analysis of phage genomes from which spacers originated (termed protospacers) revealed that they were not selected randomly [6]. Detailed analysis has found a short specific sequence called PAM, which is present in the target DNA [34,45], but is not integrated into the host CRISPR array. This suggested that PAMs help differentiate between the required DNA target (invader) and self-DNA (e.g. CRISPR loci) [27,45,46]. PAMs are typically 2–5 nt long, and they are required at both the adaptation and interference stages [47,48]. In *E. coli* PAM sequence is located upstream of the protospacer in invading DNA and the consensus sequence is 5′-AAG-3′ on the nontarget strand. In addition, protospacers with noncanonical (different) PAMs can be captured and acquired by Cas1–Cas2 complex [49–51]. Some noncanonical PAMs still support CRISPR interference, but majority fail to provide protection of cells from phage infection [49,52,53]. In interference, PAMs are used for locating and unwinding target DNA during CRISPR target recognition [28,34,48,54] and to prevent self-targeting. In adaptation, the Cas1–Cas2 complex captures DNA as protospacers by recognising PAM sequences and mediate their correct integration orientation [54–58]. To prevent self-targeting, the PAM sequences must be removed from the protospacer before integration into the CRISPR array [55,57]. Consequently, all spacers in the CRISPR array are flanked by a 5′-CCG-3′ sequence that contains the least-preferred nucleotides at each position. This repeat sequence flanking the spacer disfavors Cascade-mediated R-loop formation even with a perfectly matched spacer [34], which prevents self-targeting and protects the host.

New spacers are predominantly integrated at the leader end of the CRISPR array creating a chronological order of viral infections. Spacer integration is accompanied by a repeat duplication mediated by a staggered cleavage by the Cas1–Cas2 complex [59–61]. Selection and integration of spacers is a carefully controlled process, but occasionally host chromosomal fragments can be acquired as new CRISPR spacers [16]. Self-acquisition is expected to result in autoimmunity and programmed cell death, but lethality can be avoided by acquisition of self-derived spacers with a modified PAM, absence of effector *cas* genes or anomalous insertions of spacers [16,49–51,62]. On the other hand, to overcome CRISPR resistance, viruses and plasmids accumulate ‘escape’ mutations in the targeted protospacer or its PAM [27,58]. Cleavage of invading DNA is not initiated if there are mutations either within the PAM or the seed region of the target sequence [27,37]. This provides a simple route for the phage to escape CRISPR immune response and make host cells sensitive again. At the same time, despite inhibition of interference, some mutations in PAM sequences will stimulate rapid acquisition of new spacer targeting the same phage [51]. Therefore, the immunity can be restored by inserting new spacers in the CRISPR arrays from the same DNA. This is called priming and will be described later in the text [56,58].

The canonical PAM is highly abundant in the *E. coli* genome (there are 127,081 PAMs in the genome; [63]), and it is estimated that a Cascade spends about 30 ms in the search for a PAM [28]. However, PAM recognition in type I–E was observed to be more promiscuous in comparison with other type I or type II systems. Three peptide motifs within the Cas8e (Cse1) subunit in the Cascade complex are responsible for PAM recognition [34,64]: a glutamine wedge, a glycine loop, and a lysine finger [34]. Cse1 recognises the optimal PAM in the minor groove in dsDNA [34] and initiates dsDNA opening by inserting the glutamine wedge. This is followed by directional melting and strand invasion of crRNA and formation of a crRNA–DNA heteroduplex at the target strand. The non-target strand is actively guided ∼25 Å away from the target strand along the Cascade surface, probably as a mechanism to stabilize the seed bubble of R-loop [34]. This active guidance of non-target strand is not observed in Cas9–DNA structures [65], a likely explanation why Cascade-bound R-loop is more stable than that of Cas9 [34]. Conformational changes in Cascade accompany the R-loop formation process and reorganize the Cse1 surface which prepares Cascade for recruitment of Cas3. Depending on PAM sequences and seed mismatches, conformational change of Cse1 recruits Cas3 either for priming or DNA degradation [32].

PAM sequences are abundant in the host genome as well as being present and detected in invader elements; therefore, it is unlikely that PAMs alone provide mechanism for Cas1–Cas2 to distinguish between self from non-self-DNA. Additional help comes from replication-dependent discrimination mediated by RecBCD [66] and from interaction with interference during primed adaptation (see later in the text). However, there may be other non-Cas host proteins that participate to couple replication of the invading DNA with its capture by Cas1–Cas2. These may provide spatio-temporal control of naive adaptation [67] when interference complexes are unable to act because there is no prior immunity.

## Classification of the CRISPR-Cas systems, the structure and role of the CRISPR array in spacer integration

**Figure 1:**
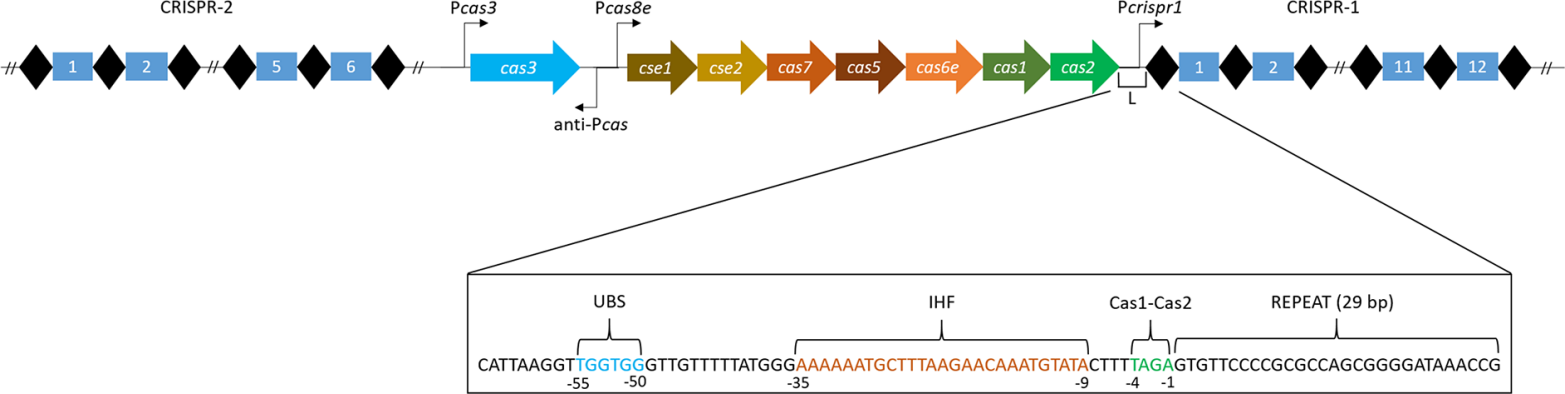
A diagram of organisation of the CRISPR-Cas Type I-E area of *E. coli* K-12 showing eight genes, four promoters, leader region, CRISPR-1, and CRISPR-2 array Sequences of repeat, UBS (upstream binding element), Cas1-Cas2, and IHF binding sites is as described in [86]. For details see the text.

*E. coli* K-12 belongs to the Class 1 type I-E CRISPR-Cas system which consists of seven genes organized in a single operon (*cse1* or *cas8e*, *cse2*, *cas7*, *cas5*, *cas6e*, *cas1*, and *cas2*), the separate signature gene *cas3*, the CRISPR-1 array containing 12 spacer-repeat units (Figure 1), and CRISPR-2 with six spacer-repeats [68,69]. CRISPR-1 and -2 have the same repeat sequence of 29 bp and 95% of spacers are 32 bp long, varying between 31 and 34 bp [3,16,68,70]. The size of the protospacer is 33 bp, but the last nucleotide of the new repeat is derived from the first nucleotide of the incoming spacer, which is the last nucleotide G of the 5′-AAG-3′ PAM sequence [56,58,60]. This guanine nucleotide was originally considered to be a part of the repeat sequence, hence there are differences in the size of the repeat/spacer in the literature. Independent promoters control transcription of Cas components (P*cas3*, P*cas8e*, P*crispr1*, and P*crispr2*), which are under normal growth conditions silenced by the activity of non-Cas host factor H-NS protein, a global transcriptional repressor [19,69,71–73], or by StpA (H-NS paralogue) at P*cas8e* when H-NS is absent [74]. The divergent promoter, anti-P*cas*, is located on the opposite strand and is also repressed by H-NS [69]. Its role is unknown and it is suggested to interfere with transcription termination or translation of the *cas3* gene [69]. Transcription from the P*cas8e* promoter is also repressed by the cAMP-dependent protein CRP, and activated by LeuO, a pleiotropic positive regulator of many genes [71,74,75]. H-NS and LeuO compete for the same DNA-binding sites, and have been suggested to act as a stochastic genetic switch in individual cells [76]. H-NS is in advantage since it is present at constant levels throughout the growth cycle, while LeuO is transiently expressed and is also repressed by H-NS [76,77]. This is probably the reason why the *E. coli* CRISPR-Cas system is inactive defence mechanism under laboratory growth conditions and spacers mostly do not match sequences in well-known phages or plasmids [78,79]. The cellular conditions known to induce *leuO* expression, such as guanosine tetraphosphate (ppGpp), amino acid starvation, or stationary growth phase unfortunately do not induce CRISPR-Cas [71,73]. Similar type of CRISPR-Cas regulation by H-NS is observed in S. Typhi IMSS-1 and *K. pneumoniae* NTUH-K2044, but it is unclear whether H-NS is a general transcriptional repressor of CRISPR-Cas in *Enterobacteriaceae* [42,80]. The reason why the CRISPR-Cas system in *E. coli* is silenced is not known, but it could be that constant expression of *cas* genes might be harmful to the cell [69]. The conditions that relieve H-NS silencing after phage or foreign DNA challenge have yet to be determined.

However, when spacers are inserted by engineering or are acquired naturally in the CRISPR array, and the CRISPR-Cas system is activated by an *hns* mutation or overexpression of the transcription factor LeuO, the CRISPR-Cas immunity is functional, but is temperature-dependent. It is active at 30°C, but inactive at 37°C due to the inhibition of Cas3 nuclease activity [19,71,72,74], so incubation temperature was proposed to be a switch that regulates CRISPR-Cas immunity in *E. coli* [73,81].

The CRISPR-1 array is preceded by an AT-rich non-coding leader region of roughly 500 bp, which contains a transcription start site about 50 nucleotides upstream from the first repeat. Transcription is controlled by a weak σ^70^ promoter located in the leader sequence, which is only partially controlled by H-NS, so transcription of the pre-CRISPR RNA *in vivo* is not completely silenced [69]. For CRISPR-2 array, a likely transcription start site is located about 95 nucleotides upstream, and this array is also transcribed *in vivo* in an H-NS dependent way [69]. In contrast, transcription from the P*cas8e* promoter, which controls the expression of the whole operon, including the *cas1* and *cas2* genes, in *wt* cells is strongly regulated by H-NS and LeuO [71]. Two putative σ^32^ promoters have been identified by genome-wide analyses, one within the *casC* gene and the other within the *cas1* gene [82,83], but their roles in transcription of *cas1* and *cas2* in *hns*^+^ cells *in vivo* have not been confirmed to our knowledge.

Two modes of adaptation have been observed in *E. coli*. Naïve or *de novo* adaptation occurs during an initial infection so sequences that have not been encountered before are integrated [67]. In primed adaptation, a pre-existing spacer promotes spacer acquisition from the same invader sequences [58]. Induced levels of *cas1* and *cas2* and the single repeat were shown to be sufficient for naïve spacer integration [16]. New spacers are integrated at the leader proximal end of the CRISPR array where specific upstream leader DNA elements are essential for spacer integration [16,61]. This suggested that the leader has a direct role in spacer integration [16]. However, the *in vitro* results revealed that the Cas1–Cas2 complex alone integrates spacers in supercoiled DNA at all CRISPR repeat sequences and also at sites outside of the CRISPR array which was in contrast to the results *in vivo* [17]. A later study found an additional non-Cas protein, integration host factor (IHF), which acts as a specificity factor for site-specific spacer integration [84]. IHF is a heterodimeric histone-like protein that exhibits sequence-specific binding at defined regions [85]. Consensus IHF binding site is located at positions −9 to −35 of the leader region, upstream from the Cas1–Cas2 binding site (positions −1 to −4) which is upstream from the leader-repeat junction (Figure 1) [84,86,87]. DNA binding by IHF induces a DNA bending of 180° which brings the upstream DNA elements (positions −50 to −55) into contact with the leader-proximal bound Cas1−Cas2 complex, an interaction that ensures specificity and efficiency of integration [84,86,87].

## Protospacer capture by Cas1–Cas2 complex and integration

Cas1 and Cas2 form a twofold symmetric ‘a crablike complex’ consisting of two asymmetric dimers of the metal-dependent DNase Cas1 that are linked by a central Cas2 dimer [61,88]. This stable complex functions as an integrase required for spacer acquisition *in vivo* where two Cas2 proteins have no catalytic activities, only structural and integrase activity is from Cas1 [61,89,90]. In early studies, *in vitro* Cas1 was shown to cleave single-stranded (ss) DNA, double-stranded (ds) DNA, cruciform DNA, and branched DNA in a sequence-independent manner and thus was mistakenly suggested to be involved in DNA repair and recombination [91]. The crystal structure of *E. coli* Cas1–Cas2 bound with dual-forked DNA revealed that four Cas1 proteins are organized in two asymmetric Cas1 dimers, Cas1a/Cas1b and Cas1a′/Cas1b′, and Cas2 in one symmetric dimer (Cas2 and Cas2′) [88]. The optimal substrate (prespacer) for *in vitro* integration is dual-forked DNA that consists of a 23 bp duplex with two 5 nt single-stranded 3′ overhangs [88,90]. The central segment of the duplex lies on the surface of the Cas2 dimer, while the 3′ overhang inserts into the C-terminal domain of Cas1a and threads through the catalytic site. Only one Cas1 in each dimer catalyses spacer integration [88,90]. The duplex length is determined by the property of the Cas1–Cas2 complex, the distance between the two Tyr22 residues of Cas1a and Cas1a′ subunits [88,90]. The PAM-complementary sequence (5′-CTT-3′), located within the 3′ overhang, is recognized in a sequence-specific manner and is cleaved by Cas1a [88]. Cas1b and Cas1b′ subunits make contacts with the Cas2 dimer and are responsible for Cas1–Cas2 formation [88]. The 5′ overhangs do not interact with Cas1–Cas2. They are exposed to the solvent and likely trimmed by host exonucleases *in vivo* (see later in the text; [42,88]).

**Figure 2:**
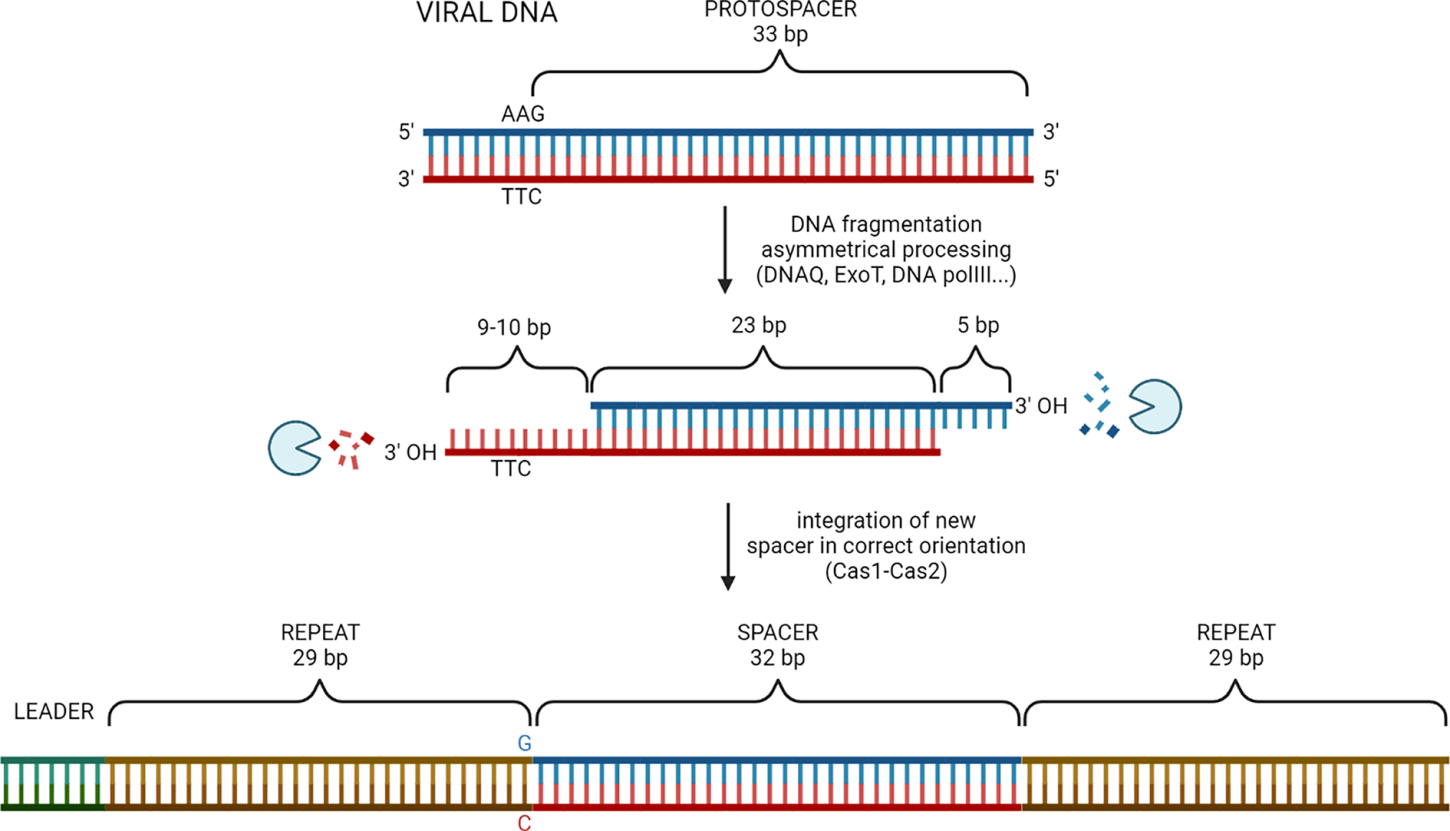
Model for asymmetrical processing and integration of the optimal spacer precursor into the CRISPR array After Cas1–Cas2 complex has made an dsDNA spacer precursor from annealed ssDNA fragments, the non-PAM 3′ overhang is trimmed to the canonical size of 5 nt by different ssDNA exonucleases, while the 3′ PAM-end is protected from degradation by Cas1 and trimming is stalled at a 9 or 10 nt 3′ overhang. This generates an asymmetrical intermediate. Adaptation complex integrates the processed non-PAM end first at the leader side of the first repeat, followed by integration of the PAM-end at the spacer side. The PAM is removed before integration of the PAM-end into the CRISPR array. The nucleotide G of the new repeat is derived from the last nucleotide of the 5′-AAG-3′ PAM sequence.

When substrate binding preferences were analyzed, the Cas1–Cas2 complex was shown to stably bind fork and other branched DNA, as well as partially dsDNA with long 3′ and 5′ overhangs *in vitro* [50,90,92]. In integration assay, ssDNA and substrates with 5′ overhangs were poor substrates for integration, and the most preferred protospacer DNA for *in vitro* integration consists of five overhanging nucleotides on each 3′ end [90], which deviates from the completely dsDNA 33 bp substrate [90]. Genetic analysis also implied that DNA duplexes with 5′ overhangs promote naïve adaptation *in vivo*, but unfortunately the results for 3′ overhangs were obstructed with the instability of the Cas1, Cas2 expressing plasmid [50]. This apparent paradox in the substrate binding preferences was recently resolved. Using single-molecule and biochemical assays, it has been found that the Cas1–Cas2 complex selects PAM-containing precursor DNAs with a long 3′ overhang from various sources of DNA, including ssDNA and partially duplexed DNA *in vitro* [57,93]. After Cas1–Cas2 captures PAM-containing ssDNA fragments, it facilitates the pairing with complementary ssDNA fragments into a precursor protospacer [57]. Upon selection of these long substrates, non-PAM 3′ overhangs are trimmed into the canonical size of 5 nt either by the Cas1–Cas2 complex itself (Figure 2) [88], or by non-Cas proteins such as DNA pol III (core or holoenzyme), DnaQ, ExoT, or other host 3′→5′ ssDNA exonucleases [55,57]. The PAM-containing 3′ end is only partially trimmed (to a size of about 8 nt) because it is protected from nucleases by the C-terminal tail of Cas1b [57].

Both 3′-OH ends of the incoming protospacer are involved in the nucleophilic attack on the CRISPR array. First, Cas1–Cas2 catalyses the integration of the processed non-PAM end at the leader site and the first repeat junction (assisted by IHF bending), which results in a half-site integration intermediate [17,89]. The second nucleophilic attack takes place at the junction of the repeat and the first spacer. There the C-terminal tail of Cas1 exposes the PAM end for ‘delayed PAM trimming’ before integration of the PAM-end into the CRISPR array [57]. However, it is not clear whether the 5′-TT-3′ sequence of the PAM is removed by Cas1 or DnaQ [55]. Thus, delayed PAM trimming of the 3′-overhang ensures correct integration of spacers in the CRISPR array [55,57].

This *in vitro* model is supported by the *in vivo* finding that spacer precursors with a characteristic asymmetric structure (5-nt overhang on the PAM-derived end and a blunt end on the non-PAM end) were detected in *E. coli* cells undergoing robust primed adaptation [94]. How are precursor DNAs with long 5′-overhangs processed is not known, but they could be degraded either by the Cas1–Cas2 complex [50] or RecJ [95] or other nucleases. After integration, the spacer is flanked by ssDNA gaps from repeat sequences that are filled through DNA synthesis by the non-Cas repair protein DNA pol I [92].

## Protospacer precursor selection by naïve and primed adaptation

Spacer acquisition by naïve or primed adaptation share all steps of DNA processing and integration into the CRISPR array, and differ only in the mechanism responsible for spacer precursor generation. In naïve adaptation, precursors are thought to originate from DNA intermediates that are formed during the repair of dsDNA breaks and free DNA ends by non-Cas host nucleases, whereas in primed adaptation DNA fragments are generated by the Cas3 nuclease during CRISPR interference [49,50,56,93,96].

Spacer integration by naïve adaptation in *E. coli* was found to be strongly dependent on the RecBCD enzyme [49,92]. Initial research on Cas1 showed that Cas1 can physically interact with proteins involved in DNA recombination and repair (RecBC and RuvB), resolve Holliday junctions, which implicated that Cas1 has roles in CRISPR immunity and in DNA repair [91]. Subsequent studies showed that the RecBCD enzyme is involved in the generation of ssDNA spacer precursors [49,92]. Namely, the RecBCD enzyme is normally involved in the repair of double-stranded (ds) DNA breaks (DSB) where it recognizes and binds to dsDNA ends that are generated after the stalling and breakage of replication forks [97]. After binding to dsDNA ends, the RecBCD helicase activity unwinds DNA and the RecBCD dsDNA nuclease activity degrades linear dsDNA into ssDNA molecules, predominantly degrading the 3′-terminated single strand into shorter fragments [98]. This degradation is modified when RecBCD reaches an 8 bp asymmetric sequence called a Chi site (5′-GCTGGTGG-3′) in the correct orientation, approaching from the 3′ side [98]. Regulatory Chi sequences are over-represented in the *E. coli* genome, there are 1008 Chi sites [99], and they serve to help recombination proteins re-establish chromosome replication at broken replication forks to preserve genomic integrity [97,100]. Upon Chi recognition, RecBCD pauses and starts loading the RecA protein onto the 3′-terminated strand to create a recombinogenic molecule, while degradation of the 5′-terminated strand is up-regulated [98,101].

In the absence of Chi, which are absent in many phages, RecBCD continues to degrade the phage DNA and serves an antiviral function [97]. Thus, depending on the Chi site recognition, the RecBCD enzyme can either repair dsDNA breaks or protect cells from foreign DNA. In other words, to produce ssDNA fragments from both DNA strands that can be used as spacer precursors, RecBCD must act on the linear host dsDNA ends made by a dsDNA break before reaching Chi or on free dsDNA ends of foreign DNA that lack Chi. Indeed, the majority of protospacer hotspots were mapped near the origin of replication (*oriC*), the replication terminus (*Ter*), and upstream of the CRISPR-1 array [49,50] where dsDNA breaks are more common, and to high-copy-number plasmids where replication forks are much more abundant over chromosomal DNA.

Detailed analysis also showed that protospacer hotspots were defined between the sites of stalled forks and Chi sites [49]. There was a significant asymmetry in protospacer density depending on the orientation of the Chi sequence, which was lost in *recB*, *recC*, and *recD* deletion mutants. In addition, the fraction of spacers originating from the self-chromosome was ∼10-fold higher in the *recB*, *recC*, and *recD* mutants compared with the *wt* strain, but spacer acquisition was greatly reduced in all three mutants [49]. Since the RecBC enzyme from the *recD* mutant is recombination proficient but lacks only nuclease activity [102], and showed reduced adaptation like the *recB* and *recC null* mutants, it was initially proposed that the nuclease activity of the RecBCD enzyme is important for generation of ssDNA spacer precursors [49].

**Figure 3:**
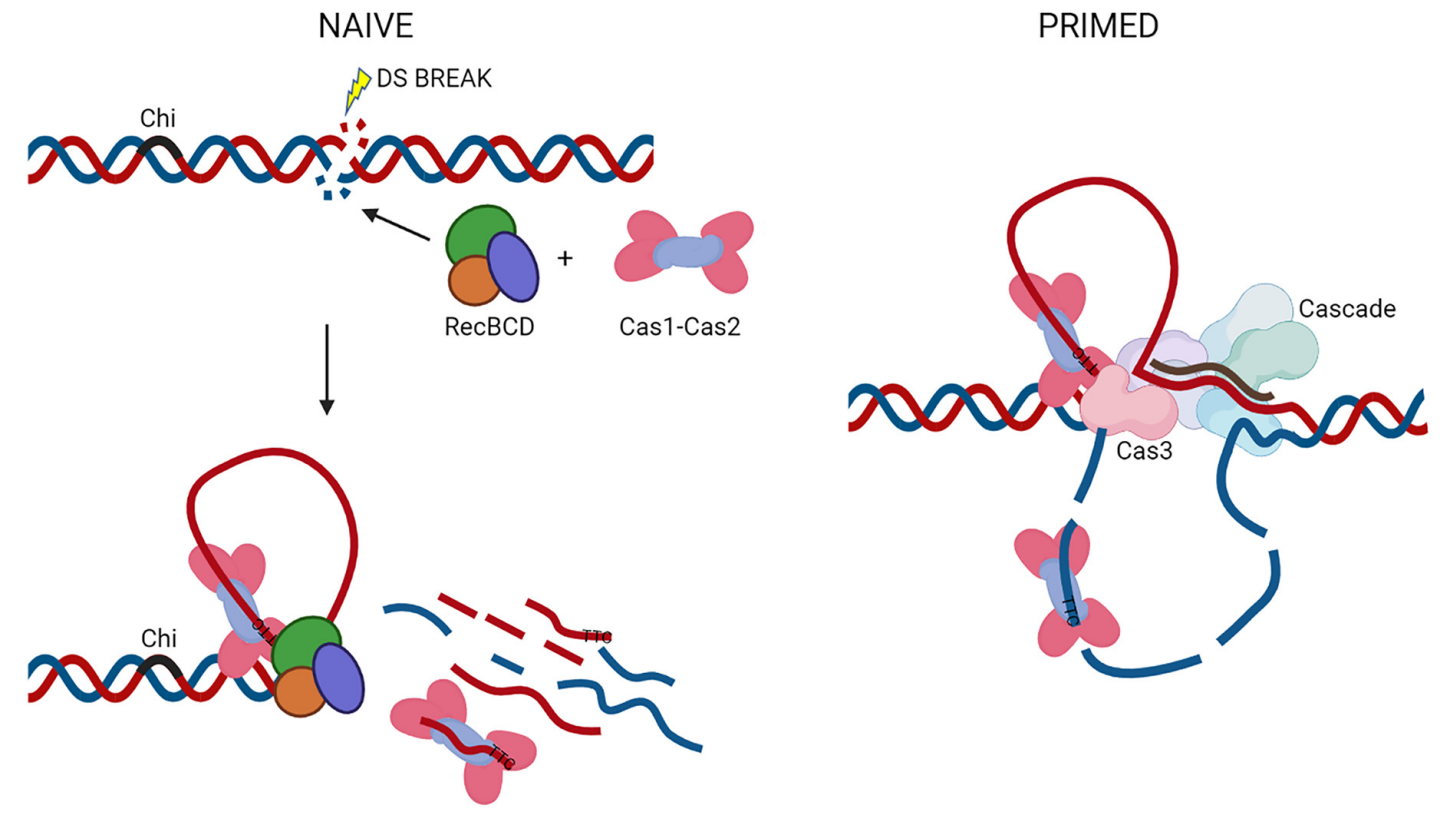
Model for ssDNA fragment capture in naïve and primed adaptation ssDNA loops and ssDNA fragments of both DNA strands are generated during RecBCD- (before Chi) and Cascade-Cas3-mediated DNA degradation. Results have suggested that RecBCD helicase activity promote naïve adaptation but it also needs assistance from host ssDNA exonucleases [50,55,57]. It is not known what is the preferred ssDNA substrate for Cas1–Cas2 complex binding *in vivo*, ssDNA loop or short ssDNA fragment. The host exo- or endo- nucleases could be required for protospacer trimming after the Cas1–Cas2 has excised ssDNA fragment, generation of ssDNA fragments prior to Cas1–Cas2 binding or both.

However, since the RecBC enzyme constitutively loads the RecA protein onto 3′ ssDNA during unwinding, without any requirement for Chi [103], this suggested that the RecA filament could also affect adaptation. Indeed, in cells lacking *recD recA*, spacer acquisition was fully restored, confirming that RecA loading by RecBC from the *recD* mutant inhibits adaptation, that the nuclease activity of the RecBCD enzyme is not required and that RecBCD helicase activity promotes naïve adaptation [50]. Separated ssDNA strands by RecBC could be nicked to short ssDNA fragments either by Cas1–Cas2 or many host ssDNA exonucleases during DNA unwinding, or taken directly from a small ssDNA loop that is generated by RecBCD before Chi (Figure 3) [50,57,104]. Since the RecBC enzyme has only one helicase motor RecB, it is not as fast as the RecBCD holoenzyme [104], and interestingly, the number of acquired spacers with a canonical PAM in the *recD recA* mutant is increased to over 80% (our unpublished results). This suggests that substrates generated in *wt* cells could be short lived which does not give Cas1–Cas2 sufficient time to identify and capture PAM-containing ssDNA fragments thus explaining the low specificity and efficiency of naïve adaptation.

In primed adaptation, protospacers are thought to derive from degradation products generated by interference [56,58,105]. In agreement, primed adaptation requires, in addition to the Cas1–Cas2 complex, also the Cascade complex, the Cas3 nuclease-helicase and a partial match between crRNA and the target DNA [52,56,58]. Primed adaptation is initiated by the recognition of the target, known as a ‘priming protospacer’. Recognition then leads to the acquisition of multiple spacers derived from the same strand as the priming protospacer [56,58,106,107]. This in contrast with other type I systems, like I-B and type I-F, where primed spacers are acquired from both strands [107,108]. This is why primed adaptation preferentially incorporates foreign genetic material compared with naïve adaptation. Intriguingly, priming can be elicited by both perfect and mismatch-containing targets that impair interference, but is also strongly influenced by the PAM variants [52,96,105,109].

PAM variants that did not support interference or those that supported rapid interference led to no or low adaptation, while those that supported attenuated levels of interference led to the highest levels of adaptation [34,105]. Priming PAM determines whether Cascade will mediate stable R-loop formation and Cas3 recruitment [34]. Recent research proposed that formation of partial R-loops and unstable Cascade binding will cause non-specific collateral ssDNA cleavage by Cas3 which can be used for priming [110]. These ssDNA fragments are assumed to be directly transferred to Cas1–Cas2 [93,110–112], which was recently confirmed by showing that the same DNA fragments of foreign DNA that will become spacers in the CRISPR array, were found associated with both Cas1–Cas2 and Cas3 in cells undergoing primed adaptation [113,114]. Since Cas3 also generates ssDNA loops in the target strand during translocation if it remains associated with Cascade [115], ssDNA loops were proposed to be important regions for Cas1–Cas2 binding (Figure 3) [57]. Due to the direct transfer of ssDNA from Cas3 to Cas1–Cas2, and association of Cas3 with Cas1–Cas2 and Cascade in the primed acquisition complex (PAC) [112,113], RecBCD is not expected to be involved in spacer precursor preparation by primed adaptation.

Indeed, RecBCD is not required in primed adaptation (adaptation was decreased), but roles for two non-Cas host helicases, the RecG and PriA helicases, have been demonstrated when cells were challenged with λ*vir* phage *in vivo* [92]. However, when a ‘self-targeting’ system was used to asses efficiency and specificity during primed adaptation, it has been found that primed adaptation and the pattern of acquired spacers was strongly affected by simultaneous deletions of nucleases *recB* and *sbcD*, and *recB* and *recJ* [95]. SbcD and RecBCD enzymes were proposed to be required for 3′ end and RecJ exonuclease for 5′ end protospacer trimming *in vivo* [95]. Very recently, using the same ‘self-targeting’ system, oligo electroporation assay, high-throughput sequencing and *in vitro* experiments, the role of RecBCD and other host proteins in primed adaptation was re-examined. It was found that RecBCD helicase and RecJ 5′→3′ exonuclease activity are involved in the 5′ end processing *in vivo* [116]. RecBCD helicase activity can be partially substituted by other host helicase such as RecQ and RecJ activity by ExoVII. On the other hand, DnaQ and ExoT were not found to be required in 3′ end processing during primed adaptation *in vivo*. In this recent study [114], a more sensitive methods were used, which can explain differences in obtained results. Yet, since primed adaptation after phage challenge probably differs from self-targeting primed adaptation, future work needs to reveal whether the same enzymatic requirements are involved in both types of primed adaptation. Apparently, non-Cas proteins appear to assist in trimming of prespacer substrates in primed adaptation.

In contrast with primed adaptation, the process of naïve adaptation is not very efficient and specific. It can acquire self-targeting spacers from the cell’s own genome and only up to 50% of newly acquired spacers are selected from protospacers associated with the canonical PAM 5′-AAG-3′ [16]. Primed adaptation is much more efficient, with >90% of acquired protospacers containing the canonical PAM and the majority originate from an area around the priming protospacer [70]. Priming also has a pronounced bias for the DNA strand from which new spacers are selected, whereas spacers acquired during naïve adaptation map to both strands of foreign DNA [56,58,70]. Also, the efficiency of priming decreases with increasing the distance from the priming protospacer [116]. The orientation of newly acquired protospacers strongly depends on their location (upstream or downstream) relative to the priming site, but the choice of protospacers does not depend on the priming protospacer orientation. Rather, the orientation of the selected spacer depends on which 3′ end strand is the Cas3 helicase domain positioned [113].

## Concluding remarks

Adaptation is a very complex and multistep process mediated by the conserved Cas1–Cas2 complex in which a molecular memory of infection is generated. This is achieved by the insertion of small DNA fragments from invading elements into the CRISPR array. In *E. coli*, two types of CRISPR adaptation have been described: naïve and primed. Both types of adaptation share the steps after the Cas1–Cas2 complex has bound to the prespacer substrate or protospacer, and differ in the process of generation of the protospacers. Throughout all steps of naïve and primed adaptation, a strong interplay with host non-Cas proteins found in type IE systems is likely to be a universal property. This strong interplay is very surprising given that the CRISPR-Cas system is silenced in *E. coli*. The roles for host proteins in CRISPR immunity were often found based on the discrepancies between *in vitro* and *in vivo* results but also from bioinformatic analysis [117]. Despite a vast knowledge gained on the mechanism of CRISPR-Cas activity in *E. coli*, we still don’t understand how and when the CRISPR-Cas system is activated under normal cell growth conditions, is it possible to observe naïve adaptation without overproduction of Cas1–Cas2 and what is the prespacer substrate, ssDNA fragment or ssDNA loop or both (Figure 3)? Is the nuclease activity of host ssDNA exonucleases required only for protospacer trimming or ssDNA fragment generation or both? We believe that future research will provide novel insights and continue to amaze us on the complexity of interactions between fighting against foreign DNA, protecting self, and maintaining genome integrity.

## Abbreviations

*cas*: CRISPR-associated genes
Cascade: CRISPR-associated complex for antiviral defence
CRISPR: clustered regularly interspaced short palindromic repeats
ds: double-stranded
DSB: double-stranded (ds) DNA break
IHF: integration host factor
*oriC*: origin of replication
PAC: primed acquisition complex
PAM: protospacer-adjacent motif
ss: single-stranded
